# Revisiting the Solubility–Permeability Relationship with Hydrophobic Drug Umifenovir in Pluronic Solutions: Impact of pH and Co-Solvent

**DOI:** 10.3390/pharmaceutics15020422

**Published:** 2023-01-27

**Authors:** Tatyana Volkova, Olga Simonova, German Perlovich

**Affiliations:** G.A. Krestov Institute of Solution Chemistry RAS, 1 Akademicheskaya str., 153045 Ivanovo, Russia

**Keywords:** solubilization, F-127 block copolymer, micelle–water partition coefficient, aggregation number, zeta potential, permeability, cellulose membranes

## Abstract

This study describes the influence of pluronic F-127 (F-127) and ethanol (EtOH) on the solubility of umifenovir (UMF) in buffer solutions of pH 2.0 and pH 7.4, and its permeability through cellulose membranes. A 44.4-fold greater UMF solubility in acidic medium as compared to an alkaline one was estimated at 310.15 K. The concentration of UMF in the saturated solution was enhanced by the interaction with F-127 micelles. The combined positive effect of EtOH and F-127 on the solubility was estimated. The aggregation number of F-127 micelles in the presence of 10% and 20% ethanol appeared to be reduced by 2.1-fold and 4.1-fold, respectively, as compared to buffer pH 7.4. The presence of ethanol in buffer pH 7.4 solution provided better solvent conditions but inhibited the formation of F-127 micelles. The impact of UMF on the aggregation number of F-127 was not pronounced and was expressed only by a slight increase of 1 and 3 units in 10% and 20% EtOH, respectively. According to the values of zeta potential, addition of EtOH reduced the stability of the system. The permeation of UMF in buffer pH 7.4 measured through the cellulose membrane MWCO 12–14 kDa was increased 1.4-fold by 10% EtOH. An increase in EtOH content to 20% reduced this effect to 1.2-fold. Decreasing effect of 1.5% F-127 on the permeability was inhibited by using 10% EtOH. The solution containing 1.5% F-127 and 10% EtOH was shown to be an advantageous system for UMF in view of the solubility–permeability balance. The authors suppose the findings of the study to be useful for the design of pharmaceutical formulations based on UMF antiviral drugs.

## 1. Introduction

**Figure 1:**
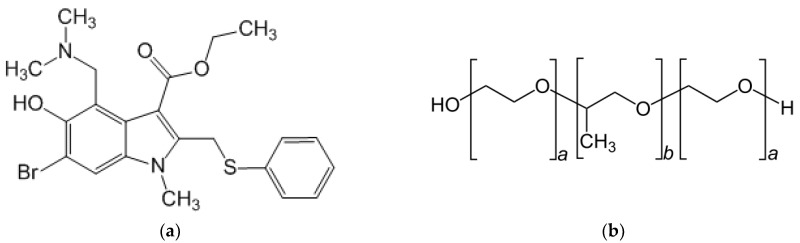
Umifenovir (UMF) (**a**) and pluronic F-127 (**b**) structures.

An indole nucleus is included in a great variety of biologically active aromatic compounds and marketed drugs. Many indole derivatives with affinities for multiple biological receptors have been synthesized, and this process is ongoing [1]. Kaushik et al. [2] reported the antiviral, anti-inflammatory, analgesic, anticancer, anti-HIV (Human Immunodeficiency Viruses), antioxidant, antimicrobial, antitubercular, anticholinesterase (towards cholinesterase enzymes), and antimalarial activity of natural and synthetic molecules containing the indole ring. Umifenovir (6-bromo-4-dimethylaminomethyl-5-hydroxy-1-methyl-2-phenylthiomethyl indole-3-carboxylic acid ethyl ester) (Figure 1a) is an indole derivative containing a benzenoid nucleus and 10 π-electrons (two from a lone pair on nitrogen and eight provided by double bonds). Umifenovir (UMF) as hydrochloride monohydrate (UMF∙HCl∙H2O) is known under the brand name arbidol—an antiviral agent demonstrating a broad spectrum of activity against flu A and B viruses and other acute respiratory viral infections (SARS)—and is widely used in Russia [3]. Moreover, taking into account the ongoing progress of coronavirus respiratory syndrome (COVID-19), trials aimed at disclosing the mechanism of UMF action against SARS-CoV-2 are shown to be of a paramount importance [4]. The authors have demonstrated UMF binding to the spike viral fusion glycoprotein of the SARS-CoV-2 Wuhan strain and its more virulent variants strain B.1.1.7 and strain B.1.351. Drug formulations based on UMF are advantageous due to low toxicity when used orally, but are moderate toxic by parenteral administration [5].

UMF in its basic form has extremely low aqueous solubility. Moreover, the hydrochloride monohydrate salt is also poorly soluble and has low bioavailability [6]. Efforts to improve the solubility of this compound have been made by several researchers. Surov et al. [7] prepared salts with pharmaceutically relevant benzoate and salicylate anions which demonstrated moderate solubility enhancement in a pH 6.8 solution. Manin et al. [8] obtained salts of arbidol (umifenovir) with maleate and fumarate anions. A new arbidol cocrystal in zwitterion form with succinic acid has also been found and characterized. The cocrystal solubility was shown to be the highest among all solid forms in both pH 1.2 and pH 6.8 solutions. Arbidol mesylate (salt) was synthesized and characterized in [9], and its apparent solubility and dissolution were greatly enhanced due to the destabilization of the drug crystal structure and strong tendency of methanesulfonic acid to form hydrogen bonds with water.

Successful attempts have been made to obtain water-soluble arbidol-based systems with lower toxicity than pure compounds using polymers, such as arabinogalactan and copolymers of acrylamide and 2-acrylamido-2-methylpropanesulfonic acid [10]. In addition, the binary and ternary arbidol hydrochloride complexes with β-cyclodextrin and polaxomer 188 [11], and solid dispersions with PEG 8000 and PEG 4000 [12] with improved dissolution have been prepared, characterized and investigated. As a result, a 13.1-fold solubility increase was achieved in kneaded ternary complex with β-cyclodextrin and 1% poloxamer 188. The percent concentrations of arbidol hydrochloride in the solid dispersions with PEG 8000 and PEG 4000 after 3 min of dissolution were 59.0 and 53.6%, as compared to a 15.4- and 14.0-fold increase of pure compound.

In the case of different micelle-forming block copolymers, for example, pluronics, solubilization of hydrophobic drugs takes place via drug interaction with the micelle hydrophobic core rather than with the hydrophilic corona [13,14]. Examples of successfully using the solubilizing agents (including pluronics) in drug formulations accompanied by simultaneous undesirable decrease of membrane permeability have been described in the literature [15,16], including in our previous studies [17,18]. It seems reasonable to select tools intended to delay the permeability reduction. For example, using penetration enhancers might solve the problem. Usually, most of the hydrophobic drugs needed for solubilization have sufficient or even high permeability, i.e., belong to the BCS class II group. Disclosing the solubility–permeability relationship can facilitate the development of the appropriate delivery system for these drugs [19]. 

Various permeation enhancers have been shown to be advantageous in pharmaceutics: fatty acids, alcohols, glycols, surfactants, sulfoxides, esters, etc. [20]. Ethanol serves many purposes in medicines as a solvent, preservative, or cutaneous penetration enhancer [21]. Meanwhile, using 40% or more ethanol in oral preparations results in a significant amount of alcohol during the course of the day [22]. Various ethanol concentrations are used in medicines: less than 2% as a solvent in the flavoring components, and at about 10% in oral liquids [23]. Systems in which both pluronic F-127 (Figure 1b) and ethanol were used in order to solubilize drug compounds have been reported in the literature [24,25,26,27]. It has been estimated that short-chain alcohols (for example, ethanol) influence the micelle formation process of block copolymers [28]. As follows, the size of the particles, aggregation, and solubilizing power towards drug compounds can vary in the multicomponent systems. Notably, the number of studies concerning permeability in the presence of block copolymers and ethanol is limited or, to be more precise, tends towards zero [29]. Deeper investigation of these processes can facilitate the design of effective delivery systems based on the micellar copolymers.

From the above review of the literature and the missed issues concerning the possibility of enhancing drug permeation using ethanol as a co-solvent in pluronic F-127 solutions, we have set forth the following objectives of our present study: (1) to disclose the influence of pluronic F-127 (F-127) and ethanol (EtOH) on the solubility of umifenovir (UMF) through quantitative parameters (efficiency of solubilization, micelle/water partition coefficient); (2) to evaluate the aggregation behavior of F-127 (particle sizes, average molecular weight of the forming micelles, aggregation number, micelle composition, zeta potential) in the presence of UMF, ethanol, and in the ternary system; (3) to reveal the influence of F-127 and ethanol on the permeability coefficients of UMF through the cellulose membrane; (4) to shed a light on the solubility–permeability interrelation by using the example of UMF; (5) to disclose the impact of pH on the solubilization and permeability of UMF.

## 2. Materials and Methods

### 2.1. Materials

Umifenovir hydrochloride monohydrate (C_22_H_28_BrClN_2_O_4_S, CAS 868364-57-2, 98%) was purchased from Sichuan Baili Pharmaceutical Co., Ltd. (Chengdu, Sichuan, China). The procedure described by Orola et al. [30] was applied to prepare the base compound. Pluronic F-127 (PEO_100_-PPO_65_-PEO_100_, CAS 9003-11-6, average molecular weight 12,600 g/mol) and the solvents: 1-octanol (purity ≥ 99%), n-hexane (purity ≥ 0.97%), and ethanol (purity 95.0%) were purchased from Sigma-Aldrich (St. Louis, USA). The reagents for the preparation of the buffer solutions, namely potassium dihydrogen phosphate (purity ≥ 99%), disodium hydrogen phosphate dodecahydrate (purity ≥ 99%), potassium chloride (purity ≥ 99%), and hydrochloric acid fixanal (0.1 mol·dm^−3^), were received from Merk (Darmstadt, Germany). Phosphate buffer pH 7.4 (I = 0.26 mol·L^−1^) was prepared using KH_2_PO_4_ (9.1 g in 1 L) and Na_2_HPO_4_·12H_2_O (23.6 g in 1 L) salts. Buffer solution pH 2.0 (I = 0.10 mol·L^−1^) was made as follows: 6.57 g of KCl was dissolved in water, 119.0 mL of 0.1 mol∙L^−1^ hydrochloric acid was added and the volume of the solution was adjusted to 1 L with water. Bidistilled water (2.1 μS·cm^−1^ electrical conductivity) was used to prepare the buffer solutions. To verify the pH values of buffer solutions, a FG2-Kit pH meter (Mettler Toledo, Greifenzee, Switzerland) graduated with pH 4.00 and 7.00 solutions was applied. The solvents and reagents were used as received without purification.

### 2.2. Methods

#### 2.2.1. Phase Solubility Study

The solubility of UMF was measured in pure buffers pH 2.0 and pH 7.4, with the additions of 1.5 w/v%, 4.0 w/v%, 5.5 w/v%, 7.0 w/v% of F-127, and in all these solutions in the presence of 10% and 20% ethanol at 310.15 ± 0.1 K by the standard shake-flask method [31]. The concentrations of F-127 were chosen purposely to avoid the formation of a gel (at approximately 20% w/w in water at 310.15 K) [32]. Since the aim of the study was to evaluate the possibility of preparation of pharmaceutical products based on UMF, the experimental temperature was selected to be as very close to the normal temperature of a healthy human. The excess amounts of the compound were put into vials containing the respective dissolution media, shaken in an air thermostat up to equilibrium (3 days, determined from the kinetic dependences), subjected to a stay of no less than 6 h to avoid supersaturation and overestimation of solubility [33], and centrifuged (Biofuge pico, Thermo Electron LED GmbH (Langenselbold, Germany) at 12,000 rpm for 20 min at 310.15 ± 0.1 K. The apparent equilibrium solubility of UMF was determined from the absorption values obtained via spectrophotometer (Cary-50 Varian, Palo Alto, CA, USA, Software Version 3.00 (339)) using the calibration curves with 2–4% accuracy. The experimental results are presented as an average of at least three replicated experiments.

#### 2.2.2. Powder X-Ray Diffraction

The powder XRD data were recorded under ambient conditions on a D2 Phaser (Bragg Brentano) diffractometer (Bruker AXS, Karlsruhe, Germany) with a copper X-ray source (λCuKα1 = 1.5406Å) and a high-resolution position-sensitive LYNXEYE XE-T detector. The samples were placed into the plate sample holders and rotated at a speed of 15 rpm during the data acquisition.

#### 2.2.3. Light Scattering Measurements

The light scattering measurements were performed using a Zetasizer Nano-ZS (Zetasizer Nano ZS, Malvern Instruments) at a scattering angle of 90°. The light source was a He–Ne gas laser which operated at 633 nm. The samples represented the clear solutions and were prepared without any filtration in order to avoid precipitation on the filter. Each experiment was repeated at least 3 times. Zeta potential was determined using the dynamic light scattering method and Smoluchowski approximation.

#### 2.2.4. In Vitro Permeability Experiments

The permeability coefficients of UMF were measured in a Franz diffusion cell of a vertical type (PermeGear, Inc., Hellertown, PA, USA) at 310.15 ± 0.1 K. The experimental setup is fully described and illustrated in our previous study [34]. All the experiments were carried out with the help of a regenerated cellulose membrane with a molecular weight cut-off (MWCO) of 12–14 kDa (Standard Grade RC Dialysis Membrane, Flat Width 45 mm) pretreated with water for 30 min and dried under air. A cellulose membrane MWCO 6–8 kDa was additionally used for several experiments. The membrane effective surface area was 0.785 cm^2^. It was placed between the donor (7 mL of the stock UMF solution) and acceptor (pure solvent without UMF—blank solution) chambers. In the case of the pH 2.0 donor solution, the blank solution at pH 7.4 was applied in the acceptor chamber in order to simulate the compound transfer to the blood plasma. Samples of 0.5 mL were withdrawn every 30 min from the acceptor chamber and replaced with an equal amount of pure buffer pH 7.4. The solutions were analyzed using a plate-type spectrophotometer (Spectramax 190; Molecular devices, Molecular Devices Corporation, California, USA) and a 96-well UV black plate (Costar). The plot of the cumulative amount of the compound (Q) permeated through the membrane of effective surface area (A) versus time (t) was constructed and the apparent permeability coefficient (P_app_) was calculated by the equation:

P_app_ = J/C_0_ (1)

where J is the flux through the membrane derived as a slope of the permeation plot (J = dQ/(A·dt)), and C_0_ is the UMF concentration in the donor solution. P_app_ values were taken as an average of at least 3 or more experiments. Drug concentration in the acceptor chamber did not exceed 10% of the concentration in the donor chamber, i.e., sink conditions were applied in each experimental time point.

## 3. Results and Discussion

### 3.1. UMF Solubility in the Examined Systems, Phase Diagrams

**Figure 2:**
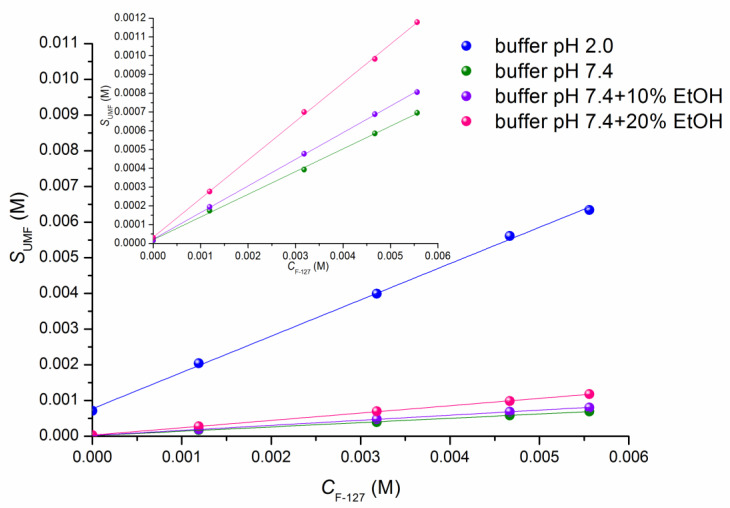
Solubility of UMF at different concentrations of F-127 (1.5–7.0%) in buffers pH 2.0 (blue) and pH 7.4 (olive) and in the presence of 10% (violet) and 20% (pink) ethanol (EtOH) at 310.15 K. Inserted plot illustrates the phase solubility of UMF in pH 7.4 and in the presence of 10% and 20% EtOH on a large scale.

In order to evaluate the solubilizing power of pluronic F-127 towards a poorly soluble UMF base, the solubility in the presence of several polymer concentrations was measured in buffer solutions of pH 2.0 and pH 7.4 using UV-spectroscopy (Figure 2, Table S1) at 310.15 K. In accordance with the pKa value equal to 6.0 [35], the compound displayed different behavior in pH 2.0 and pH 7.4 due to the basic nature of UMF: i.e., a 44.4-fold greater solubility in acidic medium as compared to alkaline one. Notably, the solubility values in pure buffers (7.11·10^−4^ M and 1.58·10^−5^ M in pH 2.0 and pH 7.4, respectively (Table S1)) measured in the present study at 310.15 K are in agreement with the literature (3.14·10^−4^ M and 8.38·10^−6^ M in pH 1.2 and pH 6.8, respectively) obtained at 298.15 K [7].

In the presence of 7.0 % pluronic F-127, the concentration of UMF in the saturated solution was enhanced 8.9-fold and 43.5-fold in buffers at pH 2.0 and pH 7.4, respectively. An essentially more pronounced solubilizing effect in pH 7.4 was determined by a higher affinity of the more hydrophobic uncharged species of UMF existing in the pH 7.4 medium to the hydrophobic core of the pluronic micelles [36]. Moreover, increasing the water–pluronic interactions upon polymer concentration increase of up to 7 % promotes structuring in water , thus inhibiting its interactions with the positively charged UMF particles at pH 2.0. From this, one can see (Figure 2) that the maximal solubility in the F-127 solution was achieved at pH 2.0 where the drug is ionized. Similarly to our results, Li et al. [37] attributed the highest flavopiridol solubility at a pH where most of the drug was ionized to the adsorption or location of the drug on the micelle–water interface when the solubility increases beyond the solubility power of the micelle hydrocarbon core. 

In the introduction section we underlined the advantages of using ethanol as a co-solvent, including in experiments in micellar systems exemplified by several drug compounds [24,25,26,27]. In this study we measured the solubility of UMF in the presence of 10% and 20% ethanol (EtOH) in pure buffer pH 7.4 and with the additions of F-127 (Figure 2, Table S1). It was shown that the solubility of UMF in the presence of 10% ethanol was only slightly higher, but was 1.8-fold greater in 20% EtOH than in buffer pH 7.4. Pluronic F-127 copolymer can be stated as a considerably stronger solvent for UMF (43.5-fold solubility increase in 7% F-127) as compared to ethanol, a weaker solvent (1.13-fold—in 10% EtOH). The EtOH molecule, consisting of an ethyl group linked to a hydroxyl group, reveals a solubilizing potential towards UMF, but this potential is essentially lower than that of F-127 due to the presence of the large hydrophobic core of the micelle. The combined effect of EtOH and F-127 was also analyzed. As a result, a 43.5-fold, 44.8-fold and 39.3-fold increase in solubility was revealed in buffer pH 7.4, buffer pH 7.4 + 10% EtOH and buffer pH 7.4 + 20% EtOH, respectively, in the presence of 7.0% F-127 (as compared to the solubility without F-127). The maximum solubility value of 1.18·10^−3^ M UMF was achieved in the (7.0% F-127 + 20% EtOH) system. This fact demonstrates that a synergistic effect of F-127 and EtOH on the solubility is more pronounced at a higher EtOH concentration. 

In order to trace possible transformations of the UMF base, the solid residuals left after each solubility experiment were isolated, dried under air and subjected to PXRD analysis together with the raw UMF base and UMF∙HCl∙H_2_O. The results are shown in Figure S1. No transformations of the UMF base were shown after the dissolution in all the systems with buffer pH 7.4. At the same time, after dissolution in the acidic buffer, the UMF base was transformed into the apparently more stable hydrochloride monohydrate (UMF∙HCl∙H_2_O) (Figure S1). This result is in accordance with the study of Surov et al. [7], in which the formation of UMF∙HCl∙H_2_O in the acidic medium was proven. Importantly, all the parameters obtained for the acidic solutions (solubility, permeability, micellization, and aggregation characteristics) refer to UMF∙HCl∙H_2_O, in spite of the fact that initially the UMF base was subjected to the experiments.

### 3.2. UMF UV-Vis Spectroscopy

**Figure 3:**
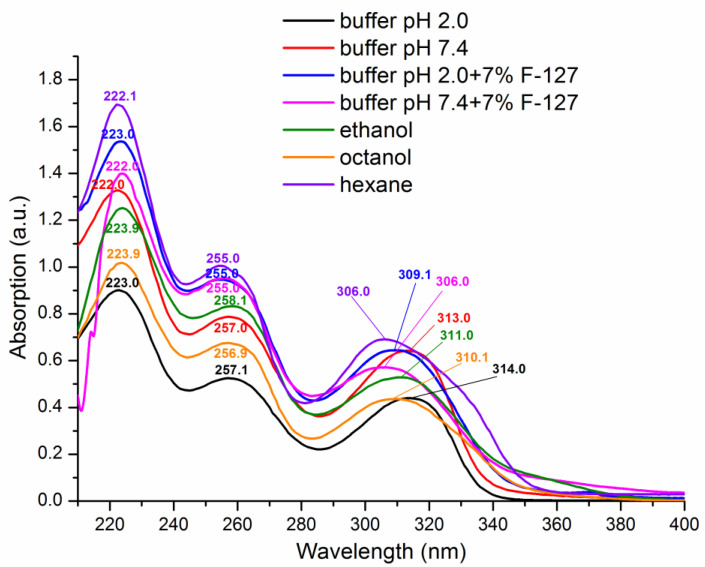
Absorption spectra of UMF in two pure buffers (pH 2.0 and pH 7.4), with F-127 (7.0 w/v%), and in organic solvents (n-hexane, ethanol, 1-octanol).

UV-vis spectroscopy was used to characterize the mode of UMF interaction with the pluronic micelles. The absorption spectra of the compound in the studied systems, together with those in the organic solvents of different polarity (n-hexane, ethanol, 1-octanol), were recoded and the wavelength maxima were detected (Figure 3).

The polarity of the solvents relative to water (equal to 1) was taken from the literature [38]: n-hexane < 1-octanol < ethanol < water. The more informative regions of the spectra were taken for analysis, i.e., 250–260 nm and 300–315 nm. Addition of F-127 to both buffers shifted the absorption maxima of UMF to that of the relatively less-polar solvents (n-hexane and 1-octanol), as shown in Figure 3, indicating the increasing affinity of the compound for the non-polar core of the micelles. At the same time, considering the region of 300–315 nm, this shift was more pronounced in the pH 7.4 medium, possibly due to a stronger tendency of the UMF base towards the non-polar core of the micelles as compared to UMF∙HCl∙H_2_O in pH 2.0, which agreed with the solubility results.

### 3.3. Determination of the Quantitative Parameters of UMF Solubilization by F-127 Micelles

To elucidate the solubilizing potential of the micelles of F-127 towards UMF, we determined the solubilizing capacity (χ) equal the amount of UMF solubilized by one mole of F-127 using the equation given in Stephenson et al. [39]:

(2) $$\chi =(S_{2}-S_{2}^{0})/(C_{polym}-cmc)=(S_{UMF}-S_{UMF}^{0})/(C_{F-127}-cmc)$$

where $S_{2}$ and $S_{2}^{0}$ are the total compound solubility at a specific polymer concentration in the solution and the intrinsic solubility, respectively, C_polym_ is the polymer (F-127) concentration, cmc is the polymer critical micelle concentration, expressed in mol∙L^−1^, and (C_polym_—cmc) is the concentration of the micelles. Sarkar et al. [40] reported the cmc values of F-127 to be essentially influenced by the presence of ethanol in the solution. The reason for this lies in the modification of the water–polymer interactions leading to different properties of complex solvent. The cmc values of F-127 in water and water/ethanol mixtures were taken from the literature [40]. Solubilizing capacities were determined from the slopes of the dependences illustrated in Figure S2. The results of the solubilizing capacity (χ) calculations are given in Table 1.

Recognizing that the solubilizing capacity in buffer pH 2.0 refers to the UMF∙HCl∙H_2_O form of the compound (not the UMF base), the comparison between the two buffers seems to be partly speculative. It can be proposed that the high solubilizing capacity of F-127 towards the monohydrate salt molecules is be due to the enhanced solubilization of the salt form of the drug in the relatively hydrophilic phase of the micelle PEO corona and aqueous solvent together [41]. Table 1 shows that the presence of ethanol in the buffer pH 7.4 solution increases the solubilizing capacity of the micelles. The promotion of the drug–copolymer interactions is, possibly, due to the dissolution of the lyotropic liquid crystalline phases formed by PEO–PPO–PEO block copolymers in water, increasing the interfacial area, swelling both PPO and PEO blocks [42], and leading to more effective solvation of the PEO block (as compared to pure water). In addition, acting as a co-solvent, ethanol can also be proposed to improve the solubility of UMF via the increased solvation by the mixed solvent [43]. 

In order to characterize the water → micelle pseudo-phase partition process, we applied the apparent micelle/water partition coefficient (K_m/w_) as a ratio between the fraction of the solute in the micelle core pseudo-phase and in the hydrophilic aqueous phase (hydrated micelle corona plus aqueous solvent) as was reported by Harris [44] and Wan et al. [45]:

(3) $$K_{m/w}=S_{m}/S_{2}^{0}=(S_{2}-S_{2}^{0})/S_{2}^{0}$$

where *S_m_* is the drug concentration in the micelle, $S_{2}$ and $S_{2}^{0}$ are the total compound solubility at a specific polymer concentration in the solution and the intrinsic solubility, respectively. The micelle/water molar partition coefficient is produced from the slope of the plot of $S_{m}$/$S_{2}^{0}$ vs. the copolymer concentration. The dependences are depicted in Figure S3 and the *K_m/w_* values are introduced in Table 1. The results (Table 1, Figure S3) showed an approximately 5.4-fold higher solubilizing potential of F-127 towards the UMF base at pH 7.4 as compared to UMF∙HCl∙H_2_O at pH 2.0, most probably due to the higher affinity of the neutral UMF base species for a relatively hydrophobic micelle core [41].

Micelle formation occurs above the critical micelle concentration and is accompanied by the removal of the hydrophobic tails of F-127 molecules from the aqueous surroundings, the association of monomers and the decrease of the free energy of the entire system. In this situation, a drug can be captured in the micelle core resulting in increased solubility and reduced free energy for solubilization. In order to quantify the apparent free energy of solubilization (transferring of the compound from the aqueous to the micellar phase) at 310.15 K, the following equation was applied:

(4) $$\Delta G_{S}^{310.15K}=-RT\cdot \ln K_{m/w}^{}$$

where $\Delta G_{S}^{310.15K}$ is the apparent solubilization free energy, *R* is the universal gas constant, and *K_m/w_* is the micelle/water partition coefficient of UMF. The values of the apparent solubilization free energy are given in Table 1. Markedly, the solubilization process is favorable in all the considered systems (negative values of $\Delta G_{S}^{310.15K}$). Expectedly, more negative $\Delta G_{S}^{310.15K}$ values were obtained in buffer pH 7.4 as compared to pH 2.0. As has been emphasized [46], depending on the solute polarity, the solubilization can occur in both the inner (PPO block) and the outer (PEO) regions of the micelles (where the substances with the intermediate polarity can be distributed along the surfactant surfactant molecules). From the data in Table 1 one can see that the addition of 10% ethanol only slightly increases the value of K_m/w_ and reduces $\Delta G_{S}^{310.15K}$. Increasing the ethanol content in buffer pH 7.4 up to 20% decreases the micelle/water partition coefficient; the driving force of this process is most likely due to disfavoring the micelle formation process since the solvent conditions are crucial for the micelle core [40]. The micelle/water molar partition coefficient serves as an indicator of the ability of the specific micellar system to be useful for drug delivery [36], and can be considered as a specific analogue of the partition coefficient in the 1-octanol/water system (logP). We checked up this proposal on the example of the UMF-buffer pH 7.4-pluronic F-127 system. The UMF distribution coefficient in the 1-octanol/buffer pH 7.4 system was calculated by the program pDISOL-X as logD^7.4^ = 4.15, which is not so far from the logK_m/w_ = 3.88 reported in our study. Moreover, the value of $\Delta G_{S}^{310.15K}$=−23.04 is also close to $\Delta G_{Distr}^{310.15K}$=−23.69.

In view of the selection of the best solubilizers for poorly soluble drugs, Malmsten and Lindman [47] have found that larger micelles are more efficient at the solubilizing process. On the other hand, Kabanov et al. [48] underlined that the size of the ideal self-assembling drug delivery system should be around 10 nm to successfully penetrate the biological tissues. From this argumentation, the sizes and aggregation numbers of the micelles in drug solutions are of special interest. Undoubtedly, among many factors (molecular weight, physicochemical properties of the drug, hydrophobicity of the micelle core, etc.), the pH and solvent composition are also crucial. In our study we determined the aggregation number of the pluronic F-127 micelles in aqueous buffers pH 2.0 and pH 7.4 (including the solutions of 10% and 20% ethanol) in the absence and presence of UMF. The aggregation number (N_agg_) was calculated by the equation [49]:

(5) $$N_{agg}=N_{F-127}=M_{w}(micelle)/M_{w}(polymer)=M_{w}(micelle)/M_{w}(F-127)$$

where *M_w_* (micelle) and *M_w_* (polymer) are the molecular weights of the F-127 micelle and F-127 monomer, respectively. The static light scattering and the Debye equation were used to determine the average molecular weight of micelles according to the literature [50]:

(6) $$\frac{H\cdot (C-cmc)}{R}=\frac{1}{M_{w}(micelle)}+2\cdot A_{2}\cdot (c-cmc)$$

Where *H* is the optical constant, *R* is the excess Rayleigh ratio at an angle of 90°, *C* is the pluronic F-127 concentration expressed in g·mL^−1^, and cmc is the critical micelle concentration in g·mL^−1^. The Debye plots for all the studied systems are illustrated in Figure S4. The composition of the micelles was estimated using Equations (7)–(9) [51]:

(7) $$\begin{array}{l} M_{w}(micelle)=N_{polymer}\cdot M_{w}(polymer)+N_{drug}\cdot M_{w}(drug)\\ =N_{F-127}\cdot M_{w}(F-127)+N_{UMF}\cdot M_{w}(UMF) \end{array}$$

where *M_w_* (polymer) = *M_w_* (F-127) and *M_w_* (drug) = *M_w_* (UMF) are the molecular weights of F-127 and UMF, respectively, *N_polyme_*_r_ = *N*_F-127_ and *N_drug_* = *N_UMF_* are the number of F-127 and *UMF* molecules in the two-component micelle. From this equation, taking into account the solubilizing capacity, the number of drug molecules can be calculated using the following equations:

(8) $$N_{F-127}=M_{w}(micelle)/(M_{w}(F-127)+\chi \cdot M_{w}(UMF)$$

N_UMF_ = χ∙N_F-127_ (9)

where χ is the solubilizing capacity. The aggregation numbers of F-127 in both the absence and presence of UMF calculated using Equations (5) and (6) are presented in Table 2. The number of the UMF molecules per F-127 micelle (Equations (8) and (9)), zeta potential, and the micelle sizes (at minimal and maximal F-127 concentrations) are also tabulated (Table 2). The combined analysis of the parameters in Table 2 allows us to disclose the overall aggregation behavior of pluronic F-127 in the studied systems.

It should be mentioned that the experimental values of pluronic F-127 aggregation numbers taken from different literature sources vary (from 3.7 [52] to 72 [53]). It seems reasonable to propose that the experimental details such as water pH, buffer components, filtration, filter type, etc. are of great significance, especially in the case of ionizable compounds [33]. Since it is difficult to provide the same filtration conditions in different labs, in this study we used carefully prepared transparent F-127 solutions without filtration in order to avoid precipitation and adsorption on the filter. Table 2 demonstrates different aggregation behavior of F-127 in the experimental media. Specifically, 2.2-fold greater N_F-127_ was determined in pH 7.4 as compared to pH 2.0. Surprisingly, increasing the number of monomer units in the content of the micelle did not change their size (r = 12.2 nm) (Table 2). Notably, the average hydrodynamic radii of the F-127 micelles in this study ranged from 5.8 to 12.2 nm; this is in agreement with the interval of 3.9–13 nm reported by Sharma and Bhatia for this copolymer [41]. Size distribution images of the micelles in all the studied systems at 1.5% and 5.5% F-127 are depicted in Figure S5. As expected, increasing the polymer concentration (from 1.5% to 5.5%) results in slightly reduced sizes only in the presence of EtOH. Lam et al. [54] have found the pluronic F-127 micelles to exhibit a core–shell structure consisting of a hydrophobic poly(propylene oxide) core and a shell comprising poly(ethylene oxide) and water. The micelles were shown to be spherical in shape up to 20 wt.% F-127, which was proven in this study by the linear Debye plots. 

Another important characteristic of micellar solution is zeta potential (Z-potential), reflecting the stability of a colloid system. Usually, nanoparticles with zeta potential values above ±30 are considered as stable. In our study the absolute values of Z-potential were below −6.97, which is characteristic for pluronic-containing micellar systems. Z-potential was shown to be approximately an order of magnitude lower in buffer pH 2.0 as compared to pH 7.4, which means stability is reduced in an acidic medium. On the whole, the slightly negative values of zeta potential obtained in the present study are characteristic of uncharged amphiphilic copolymers (such as F-127) [55]. The increase in the absolute value of Z-potential with pH (from pH 2.0 to pH 7.4) can be explained by the preferential adsorption of positive or negative ions and a decrease in the number of functional groups at the double layer surface of the micelles [56]. Possibly, as a result of the outlined processes, the reorganization of the micelles occurs without any modification in size.

It is well-known that the cmc of pluronics increases in the presence of ethanol in aqueous solution, resulting in less stable micelles [46]. Moreover, addition of ethanol increases the degree of solvation of both the core and corona of the micelles and makes a better solvent for copolymers, leading to a lower aggregation number. Reduction of the interfacial tension between the surfactant hydrophobic chains and water leads to the formation of micelles with smaller hydrodynamic radii which are more energetically favorable [57]. As expected, the aggregation number of F-127 in the presence of 10% and 20% ethanol appeared to reduce by 2.1-fold and 4.1-fold, respectively. Average values of the hydrodynamic radii of the micelles also decreased by 1.7 (10% EtOH) and 4.4 nm (20% EtOH). According to the respective values of Z-potential, the addition of EtOH reduces the stability (Table 2).

In spite of the fact that hydrophobic drug molecules are often readily incorporated in the core, increasing the micelle size [58], the impact of UMF in buffer solutions of pH 7.4 on the aggregation number of F-127 is not pronounced and expressed in only a slight increase of 1 and 3 units in 10% and 20% EtOH, respectively. Sharma and Bhatia [41] attributed such specificity to a high partition coefficient in the 1-octanol/water system (logD/logP ≥ 2.3) and molecular weight > 300 Da. This is the case of UMF (logD = 4.15/logP = 4.85; M_w_ = 477.42 Da). Similar results were reported by Thapa et al. [24] for the incorporation of curcumin in F-127 micelles. Importantly, the size of the micelles was also practically the same, and Z-potential was slightly reduced both with and without UMF. As opposed to buffer pH 7.4 solutions, in pH 2.0 along with the unchanged hydrodynamic radius and Z-potential a pronounced (almost by a factor of 7) increase of the aggregation number with UMF∙HCl∙H_2_O was shown, most probably due to the arrangement of a sufficient amount of UMF∙HCl∙H_2_O particles along the micelle–water interface, in the hydrophilic corona of the micelle. Notably, all the investigated systems were characterized by the moderate polydispersity index (PDI) from 0.241 to 0.4. 

### 3.4. Study of the In Vitro Permeability of UMF through the Cellulose Membranes

Permeability in the presence of solubilizing agents (such as triblock copolymer pluronic F-127) in solution is an important issue. The specific significance proceeds from the fact that permeability can be essentially reduced even in the case of small additions of polymer. We have an interest in our research group in understanding the effect of axillary substances on the permeability and selection of their appropriate concentrations. 

In the present study, we used a model cellulose membrane MWCO 12–14 kDa to determine the permeability of UMF in all the studied systems: pure buffer solutions pH 2.0/pH 7.4, two-component systems with pluronic F-127 and ternary systems with F-127 and ethanol. In addition we used the analogous cellulose membrane with lower MWCO 6–8 kDa in order to reveal the impact of the membrane cut-off parameters on the diffusion of the compound. We made attempts to estimate the impact of the aggregation properties on the permeation rate through the membrane, and to deepen insight into the possibility of permeability regulation.

**Figure 4:**
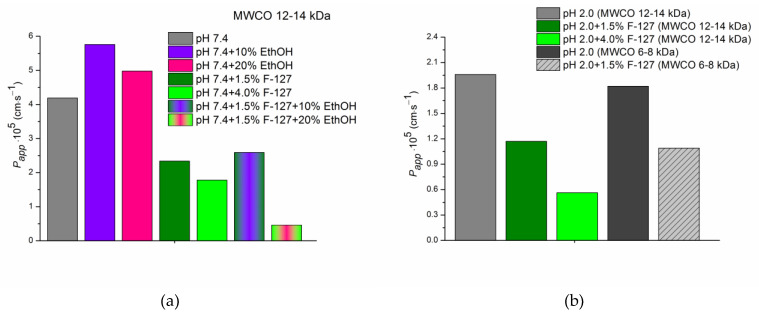
Permeability coefficients of UMF in the studied systems at 310.15 K: (**a**) pH 7.4; (**b**) pH 2.0.

Table S2 includes the experimental concentrations of the donor solutions containing UMF (C_0_) and the results of the permeation experiments: steady-state flux (J) and apparent permeability coefficient (P_app_), calculated by Equation (1). For the sake of comparison, the values of the permeability coefficients are illustrated in Figure 4 as a diagram.

**Figure 5:**
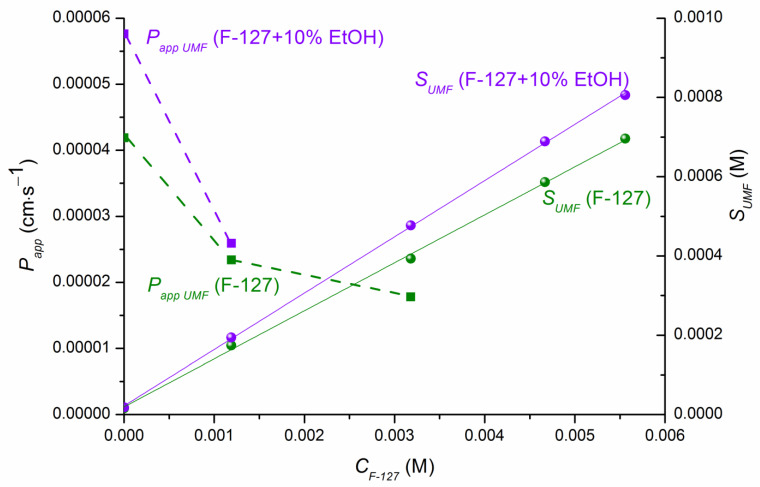
Solubility–permeability relationship exemplified by the effect of F-127 (green) and the combined effect of F-127 and 10% EtOH (violet).

Figure 4 demonstrates the impact of pH, pluronic F-127, ethanol, and the combined action of all these factors on the permeability of UMF. It can be seen that the permeability coefficient in pure buffer pH 7.4 was 2.1-fold greater than that in pH 2.0, in accordance with a higher permeation potential of the uncharged UMF base species. A trend of decreasing permeability in the presence of 1.5% and 4.0 % F-127 in both buffers was shown. Similar regularity was reported in the studies of the scientific group of Dahan, Miller, and Beig devoted to permeability of drugs in solubility-enabling formulations [19]. Approximately equal permeability reduction in both pHs upon the transition from pure buffers to 1.5% F-127 solutions was revealed, whereas for 4.0% F-127 the effect was more pronounced in buffer pH 2.0 (3.8-fold) in comparison with buffer pH 7.4 (2.35-fold) (Table S2). The influence of ethanol on the permeation rate of UMF through the membrane was exemplified by the buffer pH 7.4 systems. As shown in Figure 4 and Table S2, permeability of UMF in buffer pH 7.4 was increased 1.4-fold by 10% EtOH. Increase in EtOH content to 20% reduced this effect to 1.2-fold. A positive influence of ethanol on the passage of UMF across the membrane is to be expected, since this solvent is known as a penetration enhancer [59]. At the next step we analyzed the combined impact of F-127 and ethanol on permeability. The permeability-decreasing effect of 1.5% F-127 was slightly inhibited by using 10% EtOH. In contrast, 20% EtOH applied in combination with 1.5% F-127 contributed to further UMF permeability reduction up to P_app_ = 4.61∙10^−6^ cm∙s^−1^, that is, 9.1-fold lower than P_app_ = 4.19∙10^−5^ cm∙s^−1^ in pure buffer pH 7.4, 10.8-fold lower than P_app_ = 4.98∙10^−5^ cm∙s^−1^ in (buffer pH 7.4 +20% EtOH), and 5.1-fold lower than P_app_ = 2.34∙10^−5^ cm∙s^−1^ in (buffer pH 7.4 + 1.5% F-127). It can be concluded that the system of (1.5% F-127 + 10% EtOH) can be considered advantageous for UMF in view of solubility–permeability balance, as opposed to that with 20% EtOH concentration. The relationship between the solubility and permeability of UMF in the presence of F-127 as a solubilizing agent and ethanol as a permeability enhancer is illustrated in Figure 5.

Figure 5 visibly shows that the addition of EtOH (10%) to the system containing F-127 (1.5%) resulted not only in increased solubility but also in increased permeability growth. Markedly, in the system with 20% EtOH, an undesirable decrease of UMF permeability was evident.

### 3.5. Influence of MWCO of a Cellulose Membrane on the Permeation Rate

In order to reveal the influence of membrane MWCO on the permeability of UMF in the presence of F-127 micelles we carried out additional experiments with another membrane of the same composition but different MWCO (6–8 kDa). Permeability coefficients values of UMF in buffer pH 2.0 without and with 1.5% F-127 are given in Table S2 and Figure 4b. The P_app_ values across the membrane of MWCO 6–8 kDa were shown to be very close to the membrane of 12–14 kDa indicating the similar mode of the permeation processes. Undisputedly, in the absence of the micelles UMF passed both membranes in the same way in spite of different MWCO due to the small sizes of the UMF molecules. As it was shown in the literature, both cellulose membrane with a molecular cutoff weight of 12–14 kDa [60] and 6–8 kDa [61] are impermeable for micellar structures. Due to this fact, only “free” UMF molecules (not included into F-127 micelles) pass the membranes resulting in a negligible effect of MWCO on the permeability.

## 4. Conclusions

In the present study, the solubility of umifenovir was successfully improved with triblock copolymer pluronic F-127 and ethanol. The maximal solubility value was determined in the presence of 7.0% F-127 and 20% EtOH. A synergistic effect of the additives on solubility was estimated. The PXRD analysis demonstrated the transformation of the UMF base to an apparently more stable hydrochloride monohydrate (UMF∙HCl∙H_2_O) at pH 2.0. The quantitative parameters characterizing the solubilizing efficacy of F-127 towards UMF were determined. The higher solubilizing capacity of F-127 towards the monohydrate salt molecules as compared to the UMF base was attributed to the enhanced solubilization of the salt form in the relatively hydrophilic phase of the micelle PEO corona and aqueous solvent together. The presence of ethanol in buffer pH 7.4 solution provided better solvent conditions but inhibited the formation of the micelles. The micelle–water partition coefficient was very close to the distribution coefficient in the 1-octanol/water system. Zeta potential was shown to be approximately an order of magnitude lower in buffer pH 2.0 as compared to pH 7.4, which means the system has reduced stability in an acidic medium. The aggregation number of F-127 micelles in the presence of 10% and 20% ethanol appeared to be reduced 2.1-fold and 4.1-fold, respectively, as compared to the buffer at pH 7.4. Average values of the hydrodynamic radii of the micelles were also decreased by 1.7 (10% EtOH) and 4.4 nm (20% EtOH). According to the respective values of zeta potential, the addition of EtOH reduces the stability. The impact of UMF on the aggregation number of F-127 was not pronounced and was expressed only by a slight increase of 1 and 3 units in 10% and 20% EtOH, respectively.

The permeability coefficient of UMF in pure buffer pH 7.4 was 2.1-fold greater than in pH 2.0 in accordance with a higher permeation potential of the uncharged UMF base species. The permeation of UMF in buffer pH 7.4 was increased 1.4-fold in 10% EtOH. The increase in EtOH content to 20% reduced this effect to 1.2-fold. The permeability-decreasing effect of 1.5% F-127 was inhibited by using 10% EtOH. The solution containing 1.5% F-127 and 10% EtOH was shown to be an advantageous system for UMF in view of the solubility–permeability balance. No differences were revealed between the UMF permeability coefficients across the cellulose membranes with MWCO 12–14 and 6–8 kDa.

We hope the obtained results will enable improvements in the functioning and properties of micelle-based drug nano-systems by fine-tuning with the help of additional components such as alcohols. The findings of this study would be useful for the design of the formulations with the optimal solubility–permeability interrelation based on UMF antiviral drugs and their application in the pharmaceutical industry and research.

## Figures and Tables

**Table 1:** Solubilizing capacity (χ), apparent micelle–water partition coefficients (K_m/w_) and the free energy of solubilization ($\Delta G_{S}^{0}$ ) in the systems of UMF with F-127 in the solutions of pH 2.0 and pH 7.4 at 310.15 K.

System	Buffer pH 2.0	Buffer pH 7.4	Buffer pH 7.4 +10% EtOH	Buffer pH 7.4 +20% EtOH
χ	0.996 ± 0.025	0.120 ± 0.003	0.140 ± 0.001	0.205 ± 0.004
K_m/w_	1400 ± 36	7600 ± 205	7800 ± 72	6827 ± 118
$\Delta G_{S}^{310.15K}$ (kJ·mol^−1^)	−18.68 ± 0.37	−23.04 ± 0.46	−23.11 ± 0.46	−22.77 ± 0.46

**Table 2:** Numbers of pluronic F-127 (N_F-127_) and UMF (N_UMF_) molecules in the micelle, zeta potential (Z-potential) of pluronic F-127, and micelle size (r) with and without UMF in the studied systems.

System	N_F-127_	N_UMF_	Z-Potential (4% F-127) (mV)	r (1.5%/5.5% F-127) (nm)
	buffer pH 2.0			
F-127	18	-	−0.64	12.2/12.2
F-127 + UMF	25	25	−0.69	12.2/12.2
	buffer pH 7.4			
F-127	41	-	−6.97	12.2/12.2
F-127 + UMF	41	5	−5.69	12.2/12.2
F-127 + 10% EtOH	20	-	−6.21	10.5/10.5
F-127 + UMF + 10% EtOH	21	3	−4.81	10.5/10.5
F-127 + 20% EtOH	10	-	-	7.8/6.8
F-127 + UMF + 20% EtOH	13	3	-	6.8/5.8

## Data Availability

The results obtained for all experiments performed are shown in the manuscript and SI; the raw data will be provided upon request.

## Supplementary Materials

The following supporting information can be downloaded at: https://www.mdpi.com/article/10.3390/pharmaceutics15020422/s1, Figure S1: The PXRD patterns of: raw UMF base (black), raw UMF·HCl·H_2_O (red), solid residuals after the solubility experiments of UMF base in buffer pH 2.0 (blue) and with 7% F-127 (cyan), in buffer pH 7.4 (olive), with 7% F-127 (green), and with 7% F-127 and 20% ethanol (pink); Figure S2: Plots of $\left(S_{2}-S_{2}^{0}\right)$ on $\left(C_{F-127}-cmc\right)$ dependences at different concentrations of F-127 in buffers pH 2.0 (blue) and pH 7.4 (olive) and in the presence of 10 % (violet) and 20 % (pink) of ethanol; Figure S3: Plots correlating the UMF solubility in micallar F-127 solutions normalized by the aqueous solubility $\left(S_{2}-S_{2}^{0})/S_{2}^{0}\right)$ on F-127 concentration (C_F-127_) in buffers pH 2.0 (blue) and pH 7.4 (olive) and in the presence of 10% (violet) and 20% (pink) of ethanol; Figure S4: Debye-plots for systems of pluronic F-127 with and without UMF: buffer pH 2.0, buffer pH 7.4, buffer pH 7.4 + 10% EtOH and buffer pH 7.4 + 20% EtOH; Figure S5. Size distribution of F127 micelles: (a) buffer pH 2.0; (b) buffer pH 2.0 + UMF; (c) buffer pH 7.4; (d) buffer pH 7.4 + UMF; (e) buffer pH 7.4 + 10% EtOH; (f) buffer pH 7.4 + 10% EtOH + UMF; (g) buffer pH 7.4 + 20% EtOH; (h) buffer pH 7.4 + 20% EtOH + UMF; Table S1: UMF experimental solubility (S_UMF_) in buffer pH 2.0, pH 7.4, with addition of F-127, and 10/20% ethanol at 310.15 K; Table S2. Donor solution concentrations (C_0_), steady state flux (J), and permeability coefficients (P_app_) of UMF, 310.15 K.
