# Supplementary material for: Revisiting the Solubility–Permeability Relationship with Hydrophobic Drug Umifenovir in Pluronic Solutions: Impact of pH and Co-Solvent
Source: Pharmaceutics. 2023 Jan 27;15(2):422. doi: 10.3390/pharmaceutics15020422 (PMC9959244; doi:10.3390/pharmaceutics15020422)
Supplement: Supplementary file 1 [file pharmaceutics-15-00422-s001.zip › pharmaceutics-2182110-supplementary.pdf]

Supporting Information - content:

Figure S1. The PXRD patterns of: raw UMF base (black), raw UMF·HCl·H_2O (red), solid residuals after the solubility experiments of UMF base in buffer pH 2.0 (blue) and with 7% F-127 (cyan), in buffer pH 7.4 (olive), with 7% F-127 (green), and with 7% F-127 and 20% ethanol (pink). P2

Figure S2. Plots of $(S_2 - S_2^0)$ on $(C_{F-127} - cmc)$ dependences at different concentrations of F-127 in buffers pH 2.0 (blue) and pH 7.4 (olive) and in the presence of 10 % (violet) and 20 % (pink) of ethanol. P3

Figure S3. Plots correlating the UMF solubility in micallar F-127 solutions normalized by the aqueous solubility ($(S_2 - S_2^0)/S_2^0$) on F-127 concentration (C_{F-127}) in buffers pH 2.0 (blue) and pH 7.4 (olive) and in the presence of 10 % (violet) and 20 % (pink) of ethanol. P4

Figure S4. Debye-plots for systems of pluronic F-127 with and without UMF: buffer pH 2.0, buffer pH 7.4, buffer pH 7.4+10% EtOH and buffer pH 7.4+20% EtOH. P5

Figure S5. Size distribution of F127 micelles: (a) buffer pH 2.0; (b) buffer pH 2.0+UMF; (c) buffer pH 7.4; (d) buffer pH 7.4+UMF; (e) buffer pH 7.4+10% EtOH; (f) buffer pH 7.4+10% EtOH+UMF; (g) buffer pH 7.4+20% EtOH; (h) buffer pH 7.4+20% EtOH+UMF. P6

Table S1. UMF experimental solubility (S_{UMF}) in buffer pH 2.0, pH 7.4, with addition of F-127, and 10/20% ethanol at 310.15 K. P7

Table S2. Donor solution concentrations (C_0), steady state flux (J), and permeability coefficients (P_{app}) of UMF, 310.15 K. P7

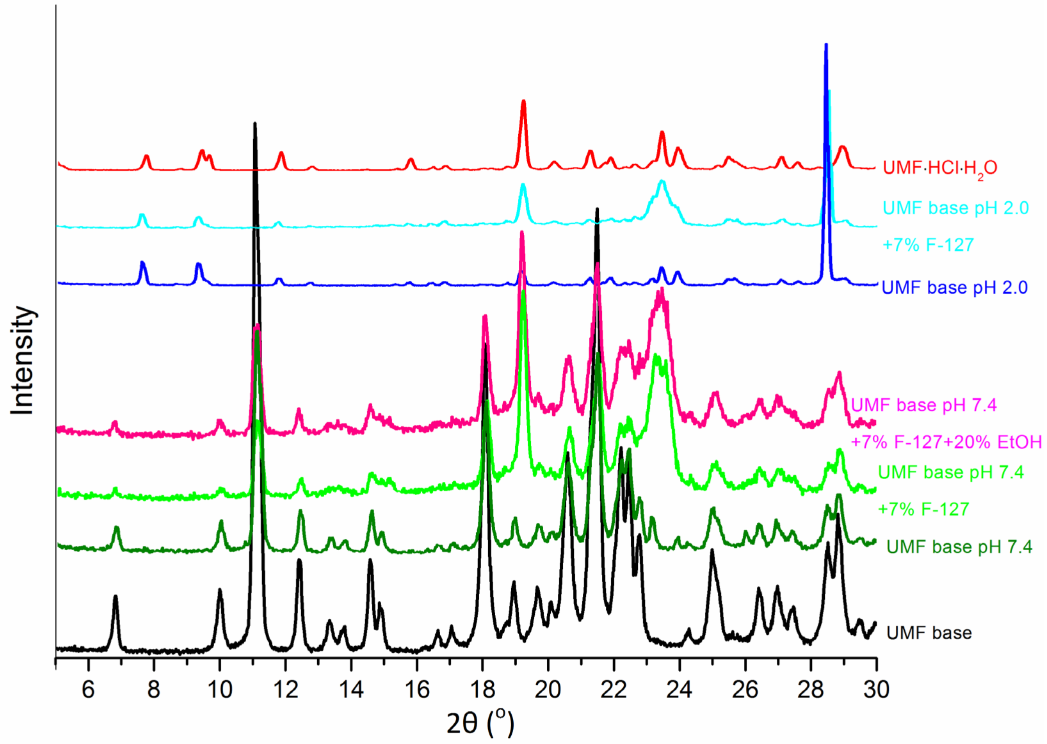

Figure S1. The PXRD patterns of: raw UMF base (black), raw UMF·HCl·H_2O (red), solid residuals after the solubility experiments of UMF base in buffer pH 2.0 (blue) and with 7% F-127 (cyan), in buffer pH 7.4 (olive), with 7% F-127 (green), and with 7% F-127 and 20% ethanol (pink).

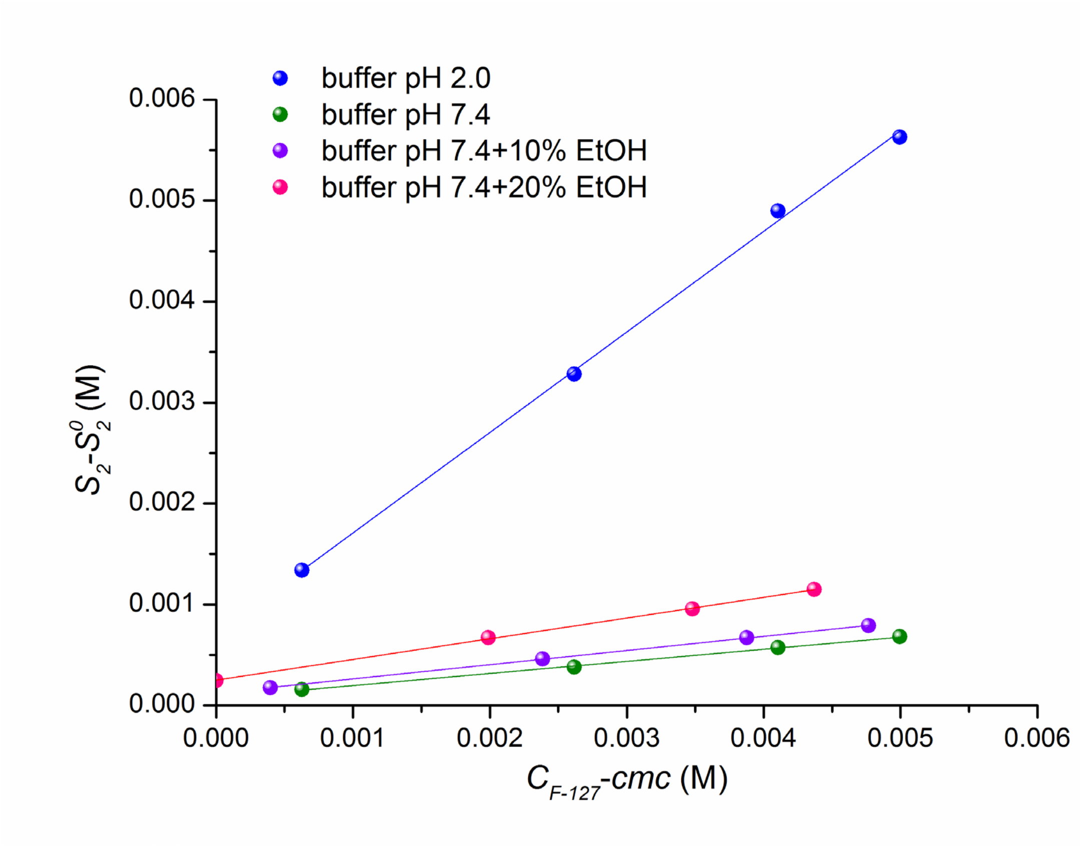

Figure S2. Plots of $(S_2 - S_2^0)$ on $(C_{F-127} - cmc)$ dependences at different concentrations of F-127 in buffers pH 2.0 (blue) and pH 7.4 (olive) and in the presence of 10 % (violet) and 20 % (pink) of ethanol.

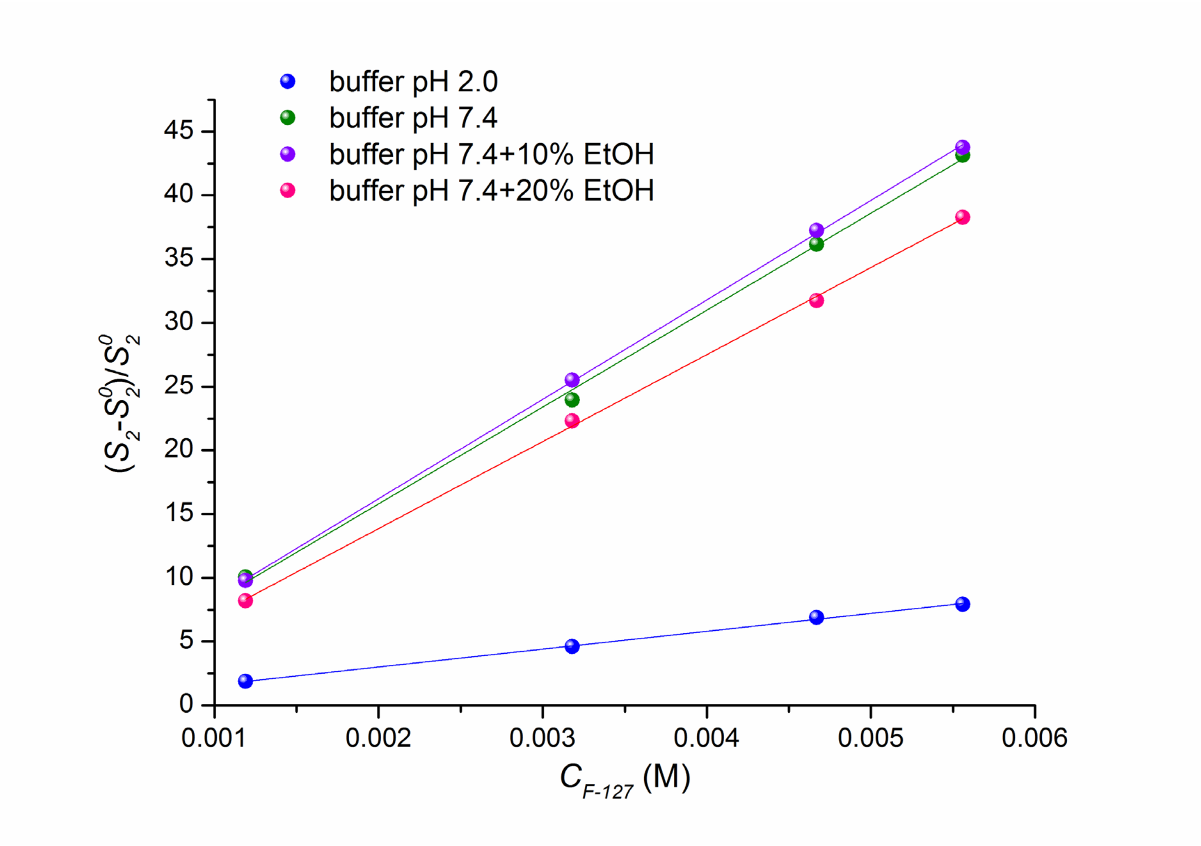

Figure S3. Plots correlating the UMF solubility in micallar F-127 solutions normalized by the aqueous solubility ($(S_2 - S_2^0)/S_2^0$) on F-127 concentration ($C_{F\text{-}127}$) in buffers pH 2.0 (blue) and pH 7.4 (olive) and in the presence of 10 % (violet) and 20 % (pink) of ethanol.

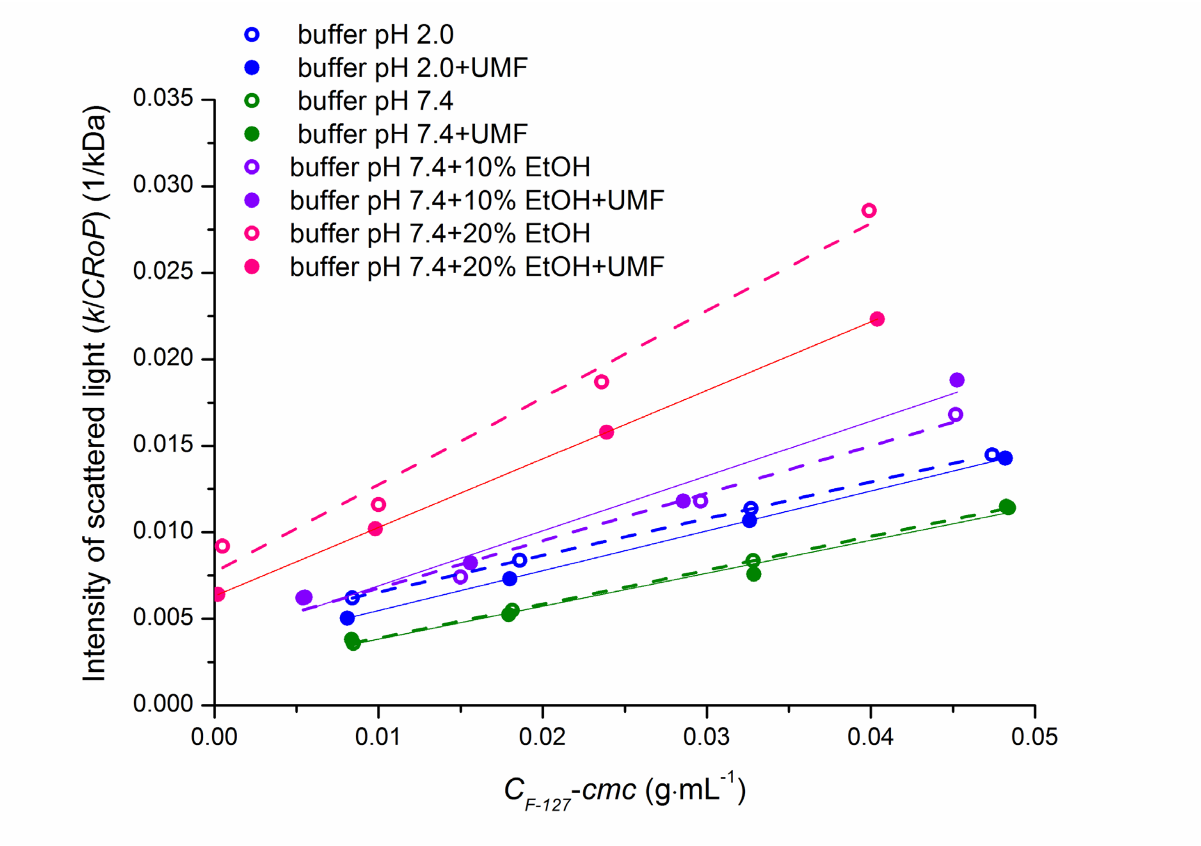

Figure S4. Debye-plots for systems of pluronic F-127 with and without UMF: buffer pH 2.0, buffer pH 7.4, buffer pH 7.4+10% EtOH and buffer pH 7.4+20% EtOH.

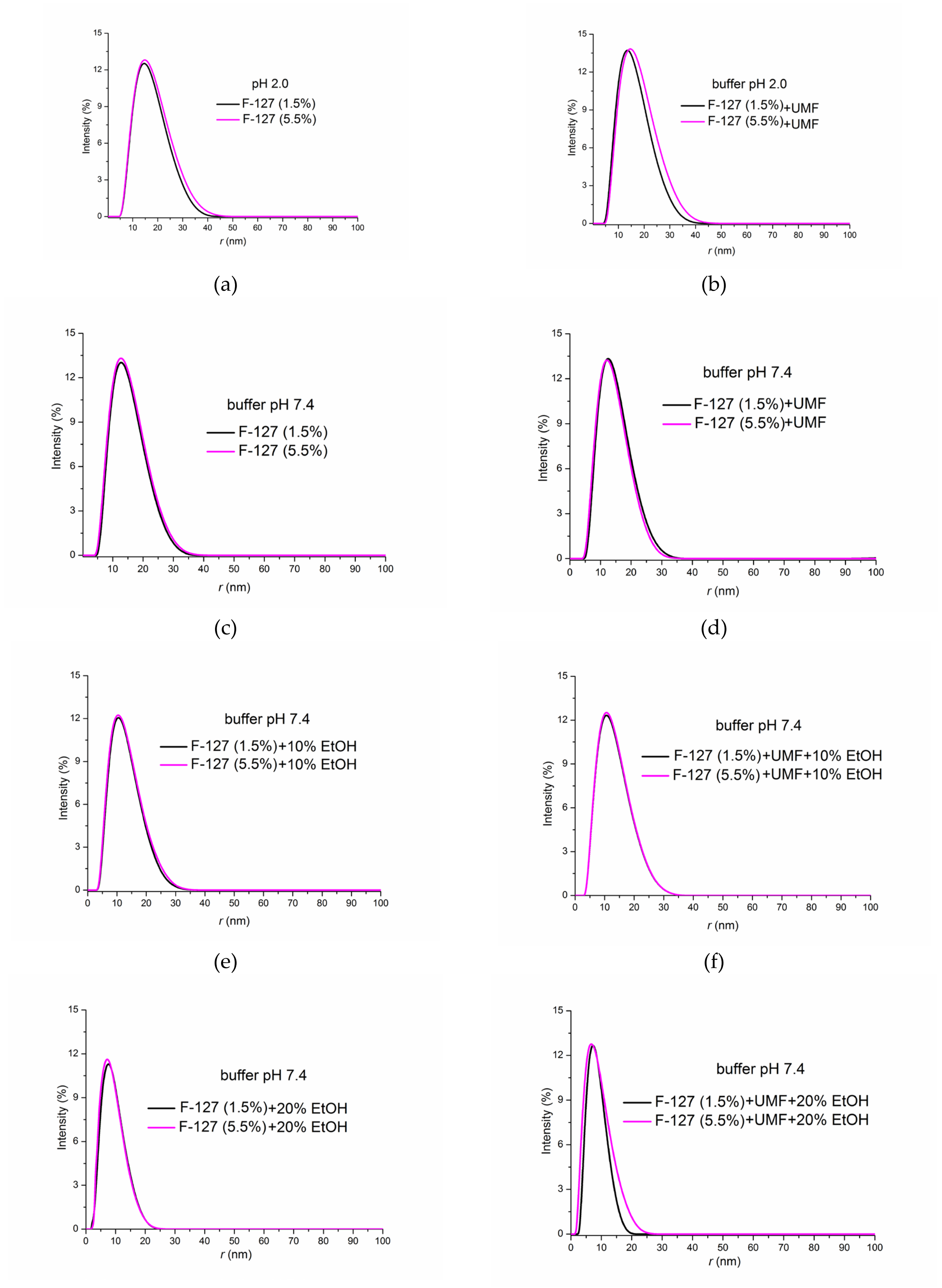

(g) (h)

Figure S5. Size distribution of F127 micelles: (a) buffer pH 2.0; (b) buffer pH 2.0+UMF; (c) buffer pH 7.4; (d) buffer pH 7.4+UMF; (e) buffer pH 7.4+10% EtOH; (f) buffer pH 7.4+10% EtOH+UMF; (g) buffer pH 7.4+20% EtOH; (h) buffer pH 7.4+20% EtOH+UMF.

Table S1. UMF experimental solubility (S_{UMF}) in buffer pH 2.0, pH 7.4, and with the addition of F-127, and 10% or 20% ethanol at 310.15 K.

C_{F-127} (w/v%)	**buffer pH 2.0**	**buffer pH 7.4**	**buffer pH 7.4 +10% EtOH**	**buffer pH 7.4 +20% EtOH**
	$S_{UMF}\cdot10^3$ (M)	$S_{UMF}\cdot10^4$(M)	$S_{UMF}\cdot10^4$(M)	$S_{UMF}\cdot10^4$(M)
0	0.71±0.02	1.60±0.01	1.80±0.01	0.30±0.01
1.5	2.05±0.04	1.74±0.03	1.94±0.01	2.76±0.03
4.0	3.99±0.08	3.93±0.03	4.77±0.03	7.00±0.05
5.5	5.61±0.04	5.86±0.02	6.89±0.07	9.83±0.11
7.0	6.34±0.03	6.96±0.04	8.06±0.07	11.80±0.09

Each solubility value represents the mean ± SD ($n \geq 3$)
The standard uncertainties are $u(T) = 0.15$ K

Table S2. Donor solution concentrations (C_0), steady state flux (J), and permeability coefficients (P_{app}) of UMF, 310.15 K.

**Investigated system**	C_0 (M)	J (μmol·cm^{-2}·sec^{-1})	P_{app} (cm·s^{-1})
	pH 7.4 cellulose membrane MWCO 12-14 kDa		
UMF	$1.04\cdot10^{-5}$	$4.36\cdot10^{-7}$	$(4.19\pm0.11)\cdot10^{-5}$
UMF+10% EtOH	$8.55\cdot10^{-6}$	$4.93\cdot10^{-7}$	$(5.76\pm0.14)\cdot10^{-5}$
UMF+20% EtOH	$1.25\cdot10^{-5}$	$6.25\cdot10^{-7}$	$(4.98\pm0.11)\cdot10^{-5}$
UMF+1.5% F-127	$5.24\cdot10^{-5}$	$1.22\cdot10^{-6}$	$(2.34\pm0.07)\cdot10^{-5}$
UMF+4.0% F-127	$5.75\cdot10^{-5}$	$1.02\cdot10^{-6}$	$(1.78\pm0.04)\cdot10^{-5}$
UMF+10% EtOH+1.5% F-127	$1.88\cdot10^{-5}$	$4.86\cdot10^{-7}$	$(2.59\pm0.06)\cdot10^{-5}$
UMF+20% EtOH+1.5% F-127	$7.77\cdot10^{-5}$	$3.58\cdot10^{-7}$	$(4.61\pm0.04)\cdot10^{-6}$
	pH 2.0 cellulose membrane MWCO 12-14 kDa		
UMF	$1.41\cdot10^{-4}$	$2.76\cdot10^{-6}$	$(1.96\pm0.07)\cdot10^{-5}$
UMF+1.5% F-127	$3.57\cdot10^{-4}$	$4.19\cdot10^{-6}$	$(1.17\pm0.02)\cdot10^{-5}$
UMF+4.0% F-127	$1.26\cdot10^{-3}$	$7.09\cdot10^{-6}$	$(5.63\pm0.18)\cdot10^{-6}$
	pH 2.0 cellulose membrane MWCO 6-8 kDa		
UMF	$1.80\cdot10^{-4}$	$3.27\cdot10^{-6}$	$(1.82\pm0.05)\cdot10^{-5}$
UMF+1.5% F-127	$3.07\cdot10^{-4}$	$3.36\cdot10^{-6}$	$(1.09\pm0.02)\cdot10^{-5}$
